# Case Report: Combining liver partition and portal vein ligation after thrombectomy for tumor isolation (CLAPT) to treat advanced hepatocellular carcinoma with portal vein tumor thrombosis

**DOI:** 10.3389/fsurg.2022.928452

**Published:** 2022-09-13

**Authors:** Zongrui Jin, Guolin Wu, Banghao Xu, Jilong Wang, Hai Zhu, Ya Guo, Minhao Peng, Tao Peng, Zhang Wen

**Affiliations:** Department of Hepatobiliary Surgery, The First Affiliated Hospital of Guangxi Medical University, Nanning, China

**Keywords:** hepatocellular carcinoma, portal vein tumor thrombosis, combining liver partition and portal vein ligation after thrombectomy for tumor isolation, associating liver partition and portal vein ligation for staged hepatectomy, future liver remnant

## Abstract

**Background:**

Primary liver cancer is the third leading cause of cancer-related deaths worldwide in 2020, and hepatocellular carcinoma (HCC) is the major pathological type. Patients with HCC complicated with portal vein tumor thrombosis (PVTT) have a poor prognosis, and controversies regarding treatment options exist among international scholars. Patients with VP4 or Cheng’s type III classification are generally considered ineligible for surgical treatment.

**Methods:**

We retrospectively analyzed three cases of HCC with PVTT who underwent a novel modified surgical procedure. The procedure included portal vein thrombectomy and portal vein ligation with liver parenchymal separation for the resection of the tumor thrombus involving the main portal vein trunk and for the isolation of the giant tumor. The three cases were then treated with targeted drugs postoperatively.

**Results:**

One case developed acute renal failure in the perioperative period, and the renal function gradually recovered after the treatment. The two remaining cases recovered uneventfully postoperatively. The prognosis of the three patients was encouraging. Only one patient died of lung metastasis after 13 months, and the remaining patients were still alive after 41 and 21 months, respectively.

**Conclusions:**

We provide a new possible surgical option for patients with advanced HCC with PVTT. The surgical procedure was inspired by associating liver partition with portal vein ligation for staged hepatectomy and portal vein thrombectomy. The survival time was significantly prolonged after the patients underwent thrombectomy, tumor isolation, and postoperative nonsurgical treatment. Hence, the combination of liver partition and portal vein ligation after thrombectomy for tumor isolation has the potential for the treatment of advanced HCC with PVTT.

## Introduction

Primary liver cancer is a common malignant tumor and has the third highest mortality rate among global malignant tumor-related diseases. Hepatocellular carcinoma (HCC) is the major pathological type (1). Most patients with HCC have reached the advanced stage after their initial diagnosis and have missed the optimal treatment time. Only about 30% of patients with HCC can receive surgery (2). HCC cells invade the portal vein system and form portal vein tumor thrombosis (PVTT) due to the anatomical characteristics of the liver blood supply system and the biological characteristics of liver cancer cells. The prognosis of patients with HCC and PVTT is generally poor, and the average median survival time is only 2.7 months (3). Surgery is the first-line treatment for HCC, but international scholars have different viewpoints regarding the suitable surgical treatment for patients with HCC combined with PVTT. The American Association for the Study of Liver Diseases (AASLD) guidelines and the Barcelona Clinic for Liver Cancer (BCLC) staging system recommend sorafenib as a standard therapy for patients suffering from HCC with PVTT (4); however, the prognosis remains poor, and the median survival is only 8.1 months (5). Compared with the conservative opinions of European and American guidelines, Asian scholars support the use of more active surgical intervention strategies (6). Associating liver partition with portal vein ligation for staged hepatectomy (ALPPS) is a new surgical operation for patients with insufficient future liver remnant (FLR) (7). ALPPS can stimulate the rapid proliferation of the remaining liver in a short period of time by changing the liver hemodynamics and *in situ* separation, leading to the safe resection of liver tumors. Therefore, ALPPS is a promising surgical treatment for HCC combined with PVTT and can bring new hope for patients while avoiding postoperative liver failure (8). Even if patients cannot undergo secondary surgery due to poor FLR hyperplasia or other reasons, the tumor isolation effect brought about by this surgery coupled with transcatheter arterial chemoembolization (TACE), tumor-targeted drugs, and other comprehensive treatment methods make the entire approach feasible (9).

We developed ALPPS, a novel modified surgical operation, by combining liver partition and portal vein ligation after thrombectomy for tumor isolation (CLAPT). The process can split the tumor by removing portal vein thrombi combined with portal vein branch ligation and liver parenchymal separation. In this study, we reported three cases of HCC combined with PVTT that were unable to undergo one-step hepatectomy due to insufficient FLR assessment but were successfully subjected to CLAPT. We described the main points of the modified technique and analyzed its feasibility, safety, and effectiveness.

## Materials and methods

### Patient base condition and preoperative evaluation

**Figure 1:**
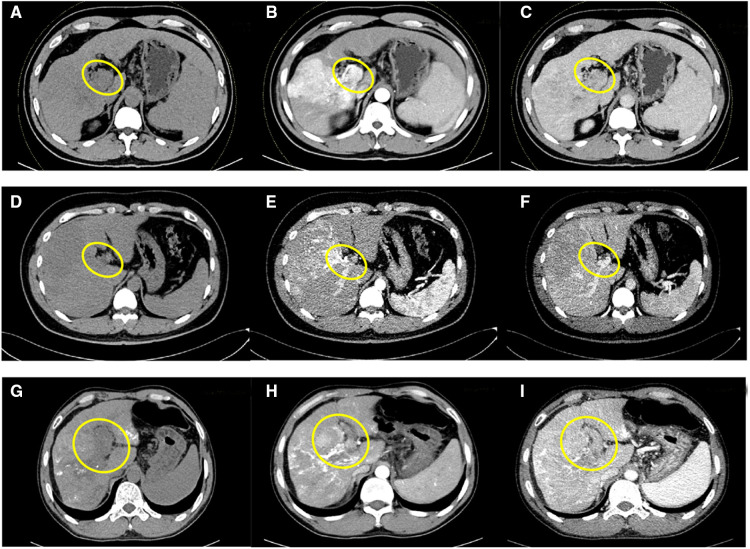
Preoperative CT images of case 1: unenhanced phase (**A**), arterial phase (**B**), and venous phase (**C**); preoperative CT images of case 2: unenhanced phase (**D**), arterial phase (**E**), and venous phase (**F**); preoperative CT images of case 3: unenhanced phase (**G**), arterial phase (**H**), and venous phase (**I**); inside the yellow circle is the portal vein tumor thrombus.

Case 1 is a 61-year-old male. His alpha fetoprotein (AFP) level was more than 20,000 *μ*g/L, and his liver function was Class A according to the Child–Pugh classification. The preoperative indocyanine green (ICG) retention 15 was 7.6%, and the FibroScan liver stiffness value was 18.3 kPa (liver elastomeric techniques). The preoperative image of the enhanced CT scan showed that the right lobe of the liver had a large, slightly low-density 10.7 cm × 10.6 cm shadow and that the main stem and right branch of the portal vein were filling defects. The tumor was mainly located in segments 6, 7, and 8 (Figures 1A–C). No extrahepatic metastases were found in other auxiliary examinations. The main preliminary diagnosis was as follows: massive HCC with right portal vein (RPV) tumor thrombus (Cheng's type III or VP4).

Case 2 is a 35-year-old male. His AFP level was 254 *μ*g/L, and the liver function was Class A. The preoperative ICG retention 15 was 4.5%, and the FibroScan liver stiffness value was 20.6 kPa. The preoperative enhanced CT imaging of the upper abdomen showed a huge mass (12.7 cm × 11.6 cm) of each segment of the right liver, a filling defect from the right branch of the portal vein to the main trunk, and a small tumor in s4 (Figures 1D–F). No extrahepatic metastasis was found. The preliminary diagnosis included the following: massive HCC with right portal vein tumor thrombus (Cheng's type III or VP4).

Case 3 is a 45-year-old man who had normal AFP and Child–Pugh Class A of liver function. The preoperative ICG retention 15 was 9.6%, and the FibroScan liver stiffness value was 16.5 kPa. The enhanced CT scans suggested the following: diffuse right liver cancer with tumor thrombus formation in the main portal vein (MPV) and left and right branches; the tumor was mainly located in the right lobe and had a maximum size of 8.8 cm; and liver cirrhosis with portal hypertension (Figures 1G–I). Extrahepatic metastasis was not observed. The preliminary diagnosis included the following: multiple liver tumors with portal vein tumor thrombosis (Cheng's type III or VP4).

The basic clinical data of the three cases are shown in Table 1. Patients with cirrhosis without portal hypertension usually require an FLR of at least 40% to minimize the occurrence of postoperative liver failure (10). Based on the CT examination, the standard liver volume (SLV) of the three cases and the proportion of the FLR were calculated using the West China formula and the IQQA-Liver system, respectively (11, 12). The results are shown in Table 1. The FLR of the three cases were obviously insufficient to complete a one-step liver tumor resection and portal vein thrombectomy, so we implemented CLAPT. This study was approved by the hospital ethical review committee, and all patients signed an informed consent form in accordance with medical ethics.

**Table 1:** Clinical information of the three cases.

Patient No.	Age	Gender	HBV	AFP (*μ*g/L)	Max Tumor Size (cm)	BCLC	Child–Pugh	MELD	PVTT classification	ICG R15 (%)	FibroScan (kp)	SLV (ml)	TLV (ml)	FLR (ml)	FLR/SLV (%)
Case1	61	Male	+	>200,000	10.7	C	A	7	Cheng's type III or VP4	7.6	18.3	1,365.52	2,063.43	471.65	34.54%
Case2	35	Male	+	254	12.7	C	A	5	Cheng's type III or VP4	4.5	20.6	1,358.23	1,995.18	315.78	23.25%
Case3	45	Male	+	2.76	8.8	C	A	8	Cheng's type III or VP4	9.6	16.5	1,239.84	1,883.21	465.18	37.52%

HBV, Hepatitis B virus; AFP, alpha fetoprotein; BCLC, Barcelona Clinic for Liver Cancer; MELD, model for end-stage liver disease; PVTT, portal vein tumor thrombus; ICG R15, indocyanine green retention rate at 15 min; SLV, standard liver volume; TLV, total liver volume; FLR, future liver remnant.

### Surgical procedure

**Figure 2:**
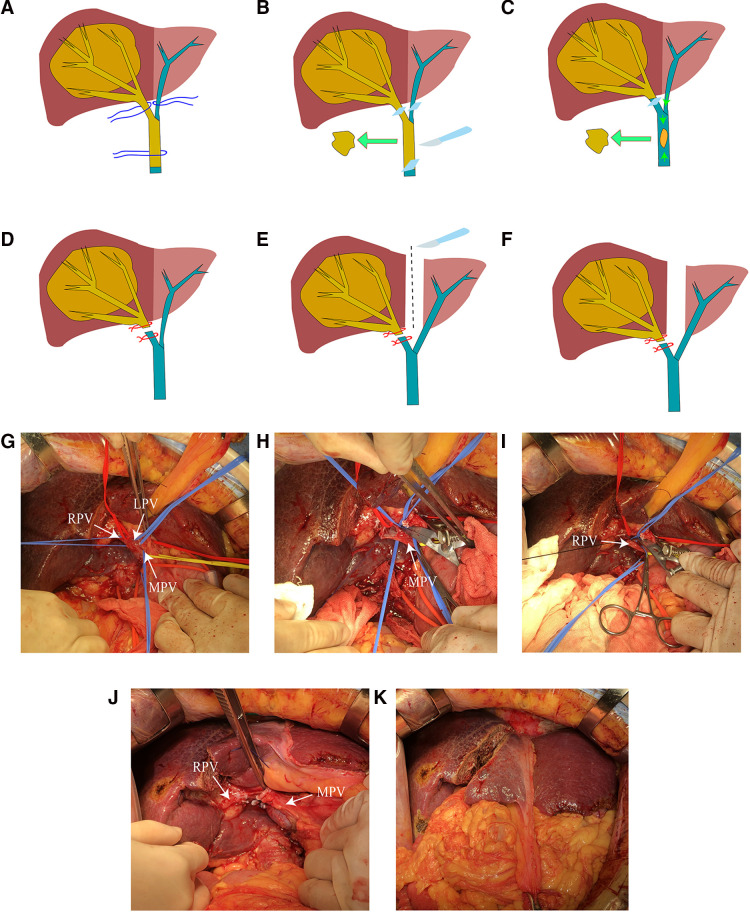
(**A**) Anatomical separation showing the LPV, RPV, and MPV. (**B**) Blocking the blood flow of the LPV, RPV, and PV and removing the tumor thrombus in the portal vein (PVTT). (**C**) Flushing out possible residual tumor thrombi by releasing MPV blood flow. (**D**) Ligation of the right portal vein stump. (**E**) Separation of the left and right liver parenchyma. (**F**) Completion of the CLAPT procedure. (**G**) Anatomical separation showing the LPV, RPV, and MPV. (**H**) Cutting the portal vein to remove the tumor thrombus. (**I**) Ligation of the RPV stump. (**J**) Separation of the left and right liver parenchyma. (**K**) Photograph of the operation completed.

Three patients were under general anesthesia when subjected to CLAPT. The patient was placed in the supine position. The surgical entry into the abdomen was layer-by-layer through the reverse “L” incision. The first hepatic portal was exposed, and the hepatic portal lymph node was cleared. The RPV distant to the bifurcation, the left portal vein (LPV), and the MPV located distal to the PVTT were occluded by vascular clip (Figure 2G). A small incision of about 1 cm was opened in the RPV or MPV. Oval forceps and thrombus removal catheter were used to remove the tumor thrombus. The PVTT in the RPV or MPV was then removed from the opening, and the possible residual tumor thrombus was flushed with portal blood flow by releasing the vaso-occlusive band of the MPV (Figure 2H). The MPV was occluded, and the residual tumor thrombus was flushed with retrograde blood by releasing the vascular occlusion band of the LPV. The portal vein cavity was flushed with heparin saline. If the tumor thrombus is tightly attached to the blood vessel wall and is difficult to peel, then the blood vessel segment can be resected, and artificial blood vessel reconstruction can be performed. After confirming that no residual tumor thrombus was present, the stump was closed by a 5-0 hemo-seal prolene. The right branch of the portal vein was disconnected, and the broken end was ligated (Figures 2I,J). After ligating the right branch of the portal vein, the left and right hepatic ischemia lines became visible. A harmonic scalpel and a cavitron ultrasonic surgical aspirator were used to split the liver parenchyma along the hepatic ischemia line, leaving the middle hepatic vein on the liver side of the tumor. The liver was split into the root of the proximal middle hepatic vein and in front of the inferior vena cava (Figure 2K). After checking the liver wound and ensuring the absence of bleeding or bile leakage, the free omentum was cropped to cover the wound. A drainage tube was then placed in the liver section and under the right diaphragm. During the operation, tumor puncture was performed to clarify the pathological results of the patients. A brief operation diagram is shown in Figures 2A–F. Postoperative monitoring of vital signs, blood routine, liver function, coagulation function, and other inspection indicators was conducted. CT and ultrasound scanning was also performed.

## Results

### Intraoperative and postoperative conditions

Based on intraoperative exploration, the liver of case 1 showed chronic liver disease and cirrhosis. The main tumor was located in the right liver, and multiple subfocals were seen around it. The right branch of the portal vein thickened, and tumor thrombi adhered to the wall and protruded to the main portal vein. Tumor thrombi were also formed on the right branch of the portal vein. For case 2, the liver had chronic liver disease and cirrhosis, and a huge mass was seen in the right lobe. Cancer thrombi adhered to the wall, from the right branch of the portal vein to the main stem. The liver of case 3 showed chronic liver disease and cirrhosis. The tumor was found in the right lobe of the liver, with subfocuses in the S3 and S4 segments. The main portal vein, the left branch, and the right branch were filled with tumor thrombi (Table 2). All three patients had normal postoperative vital signs. The patients were instructed to get out of bed 1–2 days after the surgery and have liquid food after anal exhaust. The results of blood routine, liver function, and blood coagulation function tests were rechecked after the operation (Table 3). In case 1, the number of white blood cells continued to increase after the surgery. Oliguria occurred on the fifth day after the surgery, and the continuous increase in serum creatinine was monitored. In acute kidney injury, the renal function gradually recovered after the continuous renal replacement therapy. The postoperative liver failure was graded as B, and the Clavien–Dindo classification was grade III. The postoperative levels of alanine aminotransferase (ALT) and aspartate aminotransferase (AST) increased. The abdominal drainage of POD7 reached 1,200 ml per day and improved after liver protection, diuresis, and albumin infusion. Cases 2 and 3 showed a transient increase in the ALT and AST levels after the operation; however, the levels gradually returned to normal after liver protection treatment. The postoperative liver failure was grade A, and the Clavien–Dindo complications were all grade I. The three patients underwent CT examination 2 weeks after the operation, and the FLR showed different degrees of regeneration. According to the IQQA system, the FLR of the three patients was calculated, and the SLV was determined. From case 1 to case 3, the FLR/SLV of the three patients were 53.82%, 36.36%, and 49.87%, respectively. The tumors of the three cases showed necrosis and atrophy due to the ligation of the lateral portal vein branches. Although FLR in cases 1 and 3 seemed to meet the criteria for resectability at 2 weeks after surgery, we suggest that patients should choose targeted agents along with TACE treatment considering that the patients are at an advanced stage for tumor subtyping.

**Table 2:** The three cases of intraoperative characteristics, postoperative pathological results and follow-up results.

Patient No.	Operation time (minute)	Bleeding volume (ml)	Intraoperative urine output (ml)	Pathological type	inflammation	fibrosis	Post-op treatment	tumor metastasis	Survival time (month)
Case1	390	400	1,300	Low–moderately differentiated HCC	6	6	No	No	41 (still alive)
Case2	355	100	1,550	Moderately differentiated HCC	9	6	TACE (once)	lung	13 (dead)
Case3	380	200	900	Moderately differentiated HCC	9	6	TACE (once)	no	21 (still alive)

TACE, Transcatheter arterial chemoembolization.

**Table 3:** Clinical characteristics of the three cases about preoperative and postoperative.

Patient	Time	WBC (×10^9^/L)	PLT (×10^9^/L)	ALT (U/L)	AST (U/L)	TBIL (μmol/L)	INR	Abdominal drainage (ml)
Case 1	Preoperative	7.65	284	46	65	7.1	1.05	NA
	POD 1	12.49	261	567	618	10.6	1.20	12
	POD 3	15.16	223	678	338	21.5	1.22	20
	POD 5	17.11	208	261	108	9.8	1.21	850
	POD7	20.23	213	116	92	13.1	1.23	1200
Case 2	Preoperative	6.72	163	43	57	12.3	0.99	NA
	POD 1	6.86	177	553	601	14.6	1.13	30
	POD 3	6.98	130	280	130	17.6	1.22	25
	POD 5	7.33	110	124	51	22.4	1.20	10
	POD7	5.57	154	74	46	19.1	1.21	0
Case 3	Preoperative	2.79	81	36	53	12.5	1.12	NA
	POD 1	8.68	75	173	262	32.7	1.27	35
	POD 3	8.23	71	203	192	16	1.17	80
	POD 5	5.71	92	160	122	28.6	1.16	330
	POD7	4.18	107	119	71	21.3	1.03	10

Normal reference value: WBC count (3.50–9.50) × 10^9^/L; PLT count (125–350) × 10^9^/L; ALT 9–50 U/L; AST 15–45 U/L; TBIL 3.4–21 μmol/L; INR (0.8–1.2).

WBC, white blood cell; PLT, platelet; ALT, alanine aminotransferase; AST, aspartate aminotransferase; TBIL, total bilirubin; INR, international normalized ratio; POD, postoperative day.

### Pathology and follow-up

**Figure 3:**
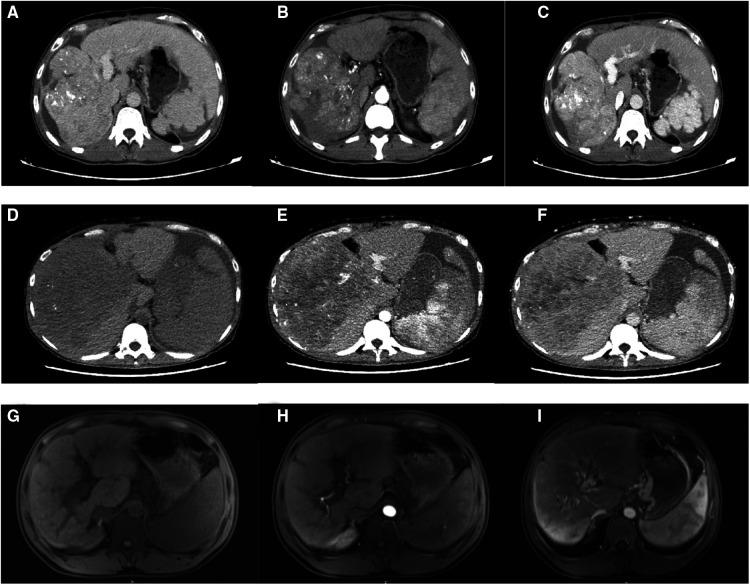
Postoperative CT images of case 1: unenhanced phase (**A**), arterial phase (**B**), and venous phase (**C**); postoperative CT images of case 2: unenhanced phase (**D**), arterial phase (**E**), and venous phase (**F**); and postoperative MRI images of case 3: unenhanced phase (**G**), arterial phase (**H**), and venous phase (**I**).

The pathology report for case 1 included the following: low to moderately differentiated HCC and Edmondson–Steiner grade III; and Ishak scores of peripheral liver tissue, with 6 points for inflammation and 6 points for fibrosis. The pathology report for case 2 was as follows: moderately differentiated HCC and Edmondson–Steiner grade III; and Ishak scores of 9 points for inflammation and 6 points for fibrosis. The pathology report for case 3 comprised the following: moderately differentiated HCC and Edmondson–Steiner grade III; and Ishak scores of 9 points for inflammation and 6 points for fibrosis (Table 2). All the three patients were treated with anti-hepatitis B drugs and administered with 0.8 g of sorafenib daily for anti-tumor treatment. Two months after discharge, the CT scan results showed no deterioration in case 1 (Figure 3A–C). Two months after hospital discharge, case 2 underwent TACE treatment. The patient then underwent CT scan 9 months after the operation (Figure 3D–F), and the result showed chest metastasis. He received conservative treatment in a local hospital and died 13 months after the operation. Meanwhile, case 3 underwent TACE treatment three months after hospital discharge. No metastasis or recurrence on MRI scan was found at 4 months after the surgery (Figure 3G–I). Tislelizumab is well tolerated as a tumor immune drug for patients with systemically treated unresectable tumors (13). Among the three cases, case 3 was treated with five courses of tislelizumab (200 mg). The two remaining patients did not receive tumor immunotherapy due to economic reasons. At the latest follow-up on March 1, 2022, cases 1 and 3 were still alive (Table 2).

## Discussion

The incidence of HCC combined with PVTT is 44%–62.2%, and the prognosis is poor (14). The important basis for treatment of HCC with PVTT is the patient's PVTT classification. The two most widely used classification systems for PVTT are the Japanese VP classification (15) and the Chinese Cheng's classification (16). No global consensus or guidelines have been established on the diagnosis and treatment of HCC combined with PVTT (17). Chinese experts suggest that when the lesion is resectable and no extrahepatic metastasis is present, patients with type I/II PVTT should undergo surgical resection of HCC as well as adjuvant TACE therapy combined with sorafenib targeted therapy after the operation. For patients with Cheng's type III or VP4 whose tumor thrombus has invaded the main portal vein, treatment can be preoperative radiotherapy or TACE and surgery (18). European and American guidelines are based on the effect of surgical intervention on the survival and quality of life of patients and recommend conservative tumor-targeted drug therapy (4).

Increasing lines of evidence show that surgical treatment can improve the prognosis of patients with HCC combined with PVTT. A literature review shows that hepatic resection is the most effective therapy for patients with HCC and PVTT (19). Roayaie et al. (20) showed that for patients with large blood vessel invasion, Child–Pugh A liver function, and BCLC C stage who underwent surgical resection, the median survival time was significantly higher than that in patients who only used drugs. The 5-year overall survival and disease-free survival rates after Vp3–4 HCC hepatectomy are equivalent to those of Vp1/2 (21). In 2016, a national research in Japan showed that the median survival time of patients with Child–Pugh A of liver function who underwent surgical treatment was 1.77 years longer than that those who did not undergo surgery. Hence, surgical treatment has benefits on the prognosis of patients with HCC and PVTT regardless of age, tumor number, liver cancer etiology, tumor marker indicators, and other factors (22). The surgical treatment for HCC combined with PVTT is selected according to PVTT classification. Patients with Cheng's type I/II may achieve radical cure by removing part of the liver or hemiliver and the invaded portal vein branch. Tumor thrombi in patients with Cheng's type III/IV can be removed through the following: (1) removal of thrombus from the portal vein in the liver section; (2) portal vein resection and reconstruction of the portal vein invaded by tumor; and (3) thrombectomy of the portal vein stump and endovascular dissection (23). Deciding whether a patient can undergo surgery requires a comprehensive assessment of resectability. Many patients with HCC are already at an advanced stage at the time of diagnosis, and some of them have PVTT and intrahepatic metastasis or massive liver cancer. These patients are often unable to receive surgical resection due to insufficient FLR but can only receive TACE and drug therapy; however, their prognosis is generally poor.

ALPPS is a new surgical operation for patients with insufficient future liver remnant. Romic et al. (24) reported a case of successful ALPPS after unsuccessful double TACE procedure, thereby confirming the superiority of ALPPS. In addition, ALPPS has been used in patients with HCC combined with PVTT and insufficient FLR. Previously reported cases were all Cheng's type I/II or Vp2–3, and patients with Cheng's type III or VP4 undergoing tumor isolation through ALPPS have not been reported. The three cases in the present study were all Cheng's type III or VP4. We combined the characteristics of ALPPS technology to thoroughly remove portal vein tumor thrombi and then proceed with the isolation and ligation of the portal vein branches of the main side of tumor. Finally, the liver parenchyma was split.

CLAPT first deals with PVTT, which can avoid the metastasis and shedding of tumor thrombus caused by intraoperative operation as much as possible. The removal of the portal vein tumor thrombus can reduce the portal vein pressure and improve the patient's liver function and life quality. Even if radical tumor resection is not possible due to insufficient FLR growth or other reasons, liver parenchymal separation and portal vein branch ligation can substantially split the tumor, thereby controlling the growth and metastasis of tumors and tumor thrombi. Peng et al. reported that three patients with Cheng's type III or VP4 underwent priority portal vein thrombus removal and hemihepatectomy and had postoperative tumor-free survival time of 13, 9, and 4.6 years (25). Hepatectomy and thrombectomy cannot only avoid acute portal vein occlusion caused by tumor thrombus but also has certain survival benefits (26). This finding provides strong evidence for our research.

Although many Asian guidelines recommend surgical resection as the preferred method for treatment of HCC combined with PVTT, it still cannot be applied to all patients due to the limitations of the scope of indications and contraindications. Our research expanded the possible application range of surgical treatment in poor PVTT classification, but we still advocate comprehensive multimethod treatment for patients with HCC and PVTT. Matono et al. (27) reported that some VP4 cases received their first surgical resection combined with focal ablation or reoperation for a longer survival times. Kojima et al. (28) also reported that patients with VP4-type HCC had prolonged survival after surgery combined with TACE treatment. TACE is the most common treatment method for patients with HCC and PVTT who cannot undergo surgery. Patients with acceptable liver function and established portal collateral circulation can benefit from TACE (29). Sorafenib is an effective molecular targeted drug that is used to treat patients with advanced HCC. Sorafenib combined with TACE can significantly prolong the survival time of patients with unresectable HCC with PVTT compared with TACE alone (30). In the treatment of advanced liver cancer, immunotherapy has become popular among scholars. Immune checkpoint inhibitors targeting PD-1/PD-L1 and CTLA4 hold great prospects (31). In recent years, clinical trials of molecular targeted drugs combined with immunotherapy for the treatment of tumors have been vigorously carried out (32).

In this study, none of the three patients received conventional surgical resection of tumors. After CLAPT treatment, a follow-up anti-tumor comprehensive treatment should be conducted. During the follow-up, cases 2 and 3 underwent TACE surgery 2–3 months after the initial surgery; all the three cases took sorafenib after surgery. The statuses of cases 1 and 3 were great, and the survival periods were 41 and 21 months, respectively. Case 2 died of lung metastasis, and the overall survival period was 13 months. The survival time of the two tumor patients was more than 1 year, which was significantly longer than the median survival time reported in the literature for VP4 HCC with PVTT treated with TACE and sorafenib (3 months, *n* = 10) (30). Although the inclusion of a large number of cases was required in the study, the preliminary follow-up results confirmed the effectiveness of the new operation combined with the comprehensive treatment plan.

We combined the ALPPS technology to expand the indications for surgical treatment of HCC combined with PVTT. The three cases all had severe cirrhosis, and the preoperative FLR was less than 40%. Case 1 had transient acute kidney injury, which was gradually relieved after the comprehensive treatment, and the other cases recovered well after operation. Therefore, CLAPT may be a new and excellent treatment option for patients with advanced HCC complicated with PVTT, but this requires more case studies. We have registered a prospective clinical trial on CLAPT in the Chinese clinical trial registry (ID: ChiCTR2200060459) to validate its safety and efficacy.

## Conclusion

We propose a novel modified surgical method for patients with advanced HCC combined with PVTT and expand the scope of surgical intervention in these patients, especially for patients with VP4 or Cheng's III type who cannot accept one-step hepatectomy. In the proposed method, the side of the liver containing the main tumor was split by removing the portal vein tumor thrombi combined with the ligation of the portal vein branch and the separation of liver parenchyma. This action contributed to the control of tumor growth and metastasis and improved the prognosis. These patients need to receive comprehensive multiple treatments postoperatively, such as immune and targeted drug therapy and TACE to further improve their survival time and quality of life.

## Data Availability

The original contributions presented in the study are included in the article/Supplementary Material, further inquiries can be directed to the corresponding author.
